# Efficacy and Safety of Different Immunosuppressive Therapies in Patients With Membranous Nephropathy and High PLA2R Antibody Titer

**DOI:** 10.3389/fphar.2021.786334

**Published:** 2022-01-17

**Authors:** Le Deng, Qipeng Huang, Jiang Wang, Kaiping Luo, Jiarong Liu, Wenjun Yan, Fang Jiang, Gaosi Xu

**Affiliations:** ^1^ Department of Nephrology, The Second Affiliated Hospital of Nanchang University, Jiangxi, China; ^2^ Department of Nephrology, The Fifth Affiliated Hospital of Jinan University, Heyuan, China; ^3^ Department of Hemodialysis, Jiujiang Hospital of Traditional Chinese Medicine, Jiangxi, China; ^4^ Department of Nephrology, Ganzhou City People’s Hospital, Ganzhou, China; ^5^ Department of Nephrology, The First Affiliated Hospital of Gannan Medical University, Jiangxi, China; ^6^ Department of Nephrology, Xinyu City People’s Hospital, Jiangxi, China

**Keywords:** membranous nephropathy, phospholipase A2 receptor, immunosuppressive therapy, remission, prognosis

## Abstract

**Background:** This study aimed to evaluate clinical features and prognosis and therapy option of patients with different risk ranks based on antibody against the M-type phospholipase-A2-receptor (PLA2Rab) level in seropositive M-type phospholipase-A2-receptor (PLA2R)-associated membranous nephropathy (MN) in a large sample size, multi-center study.

**Method:** Based on the unvalidated cut-off value of PLA2Rab above 150 RU/ml as one of the clinical criteria for high risk of progressive kidney function loss in MN according to 2020 Kidney Disease: Improving Global Outcomes (KDIGO) draft guidelines recommendation, a total of 447 patients who received cyclophosphamide (CTX) or tacrolimus (TAC) combined with corticosteroids treatment for 12 months were divided into high titer (>150 RU/ml) group and non-high titer (20–150 RU/ml) group, which were subdivided into CTX subgroup and TAC subgroup. The overall cohort was classified into CTX group and TAC group as well. Clinical parameters levels and remission rates were recorded at 3, 6, and 12 months follow-up. PLA2Rab was tested by enzyme-linked immunosorbent assay.

**Results:** Patients with high titer PLA2Rab were associated with more severe proteinuria and hypoalbuminemia compared to those with non-high titer antibody, accompanied by lower complete remission (CR) and total remission (TR) rates at 3, 6, and 12 months, which even took longer to remission. Similar remission rates differences between the two titer groups were observed in the CTX and TAC groups, respectively. PLA2Rab level at baseline was an independent predictive factor for CR and TR. In the high titer group, CR and TR rates in the CTX subgroup were significantly higher than those in the TAC subgroup at 12 months, although serious adverse events were more frequent in the former.

**Conclusion:** High-risk rank patients with PLA2Rab level above 150 RU/ml have higher disease activity and worse prognosis among patients with seropositive PLA2R-associated MN, even under different immunosuppressive therapeutic models; moreover, CTX combined with corticosteroids was preferred compared to TAC plus corticosteroids, although serious adverse events were more frequent in the former. Additionally, baseline PLA2Rab level was an independent predictive factor for clinical remission.

## 1 Introduction

In 2009, Beck et al. (2009) first discovered that M-type phospholipase A2 receptor (PLA2R) was a key target glomerular podocyte antigen, which was abundantly expressed in 70% of patients with primary membranous nephropathy (MN). Subsequent studies showed that antibody against the M-type phospholipase-A2-receptor (PLA2Rab) was found seropositivity in 57%–88.5% in primary MN (Qin et al., 2011; Ramachandran et al., 2016; Huang et al., 2017; Li et al., 2018). A cumulative number of studies have reported a relationship between PLA2Rab levels and clinical parameters, such as 24-h urinary protein and serum albumin (Hofstra et al., 2011; Kaga et al., 2019), and the detection of serum PLA2Rab might help to assess the therapeutic response (Ruggenenti et al., 2015; De Vriese et al., 2017; Guo et al., 2019; van de Logt et al., 2019), time to remission (Qin et al., 2011), prognosis stratification (Liang et al., 2019), and personalized treatment design (Glassock, 2014).

However, because the natural course of MN is long and heterogeneous, the clinical features and prognosis are highly variable. Controversy persists about the association between PLA2Rab levels with clinical characteristics and prognosis (Oh et al., 2013; Bech et al., 2014; Jullien et al., 2017; Pourcine et al., 2017). Moreover, most clinical studies were limited by small sample sizes, single-center studies, and a paucity of studies investigating the relationship according to unified PLA2Rab rank threshold. Different PLA2Rab rank cut-off values have been reported in the respective studies (Hofstra et al., 2012; Hoxha et al., 2014b; Ruggenenti et al., 2015; Ramachandran et al., 2016; Dahan et al., 2017; van de Logt et al., 2018). On the other hand, compared to Kidney Disease: Improving Global Outcomes (KDIGO) recommendations in 2012, there is an important change concerning calcineurin inhibitors (CNIs) in moderate- to high-risk patients, in which CNIs might not seem to be the best therapy for primary MN, and the most effective immunosuppressive therapy is controversial.

It is necessary to abandon the one-therapy-fits-all concept and focus on risk stratification, which included PLA2Rab level in 2020 KDIGO draft guidelines, and to identify the most effective therapeutic options in different MN subsets. The 2020 KDIGO draft guidelines recommend PLA2Rab level above 150 RU/ml as one of clinical criteria for high risk of progressive kidney function loss in MN, while the guidelines also mention that the cutoff value is not verified. To this end, we determined a risk rank threshold corresponding to the draft guidelines recommended PLA2Rab level (150 RU/ml), which help to evaluate clinical features, prognosis, and therapy option in patients with seropositive PLA2R-associated MN in a large sample size, multi-center study.

## 2 Materials and Methods

### 2.1 Study Design

A total of 447 patients received cyclophosphamide (CTX) or tacrolimus (TAC) combined with corticosteroids treatment were recruited between January 2017 and April 2021 from five Chinese nephrology centers, including the lead center—the Second Affiliated Hospital of Nanchang University, Ganzhou City People’s Hospital, Jiujiang Hospital of Traditional Chinese Medicine, the First Affiliated Hospital of Gannan Medical University, and Xinyu City People’s Hospital. The present study was approved by the Ethics Committee of the Second Affiliated Hospital of Nanchang University [No. (2016) No. 120] and conducted according to the ethical principles stated by the Declaration of Helsinki. Informed consent was obtained from all patients.

### 2.2 Patients

Inclusion criteria were as follows: (1) IMN (stage I–IV) proven by renal biopsy; (2) positive serum PLA2Rab titer at diagnosis; (3) age 18 years or more; (4) urinary protein >3.5 g/24 h, serum albumin <30 g/L, edema, and/or hyperlipidemia after 6 months anti-proteinuria treatment with angiotensin-converting enzyme inhibitor(s)/angiotensin II receptor blocker.

Exclusion criteria were as follows: (1) patients with secondary forms of MN, including autoimmune diseases, infection-related MN, and MN-related to malignancies or exposure to toxic substances; (2) immunosuppressive therapy in the last 6 months; (3) serum creatinine persistently >309 μmol/L; (4) life-threatening complications; and (5) pregnancy or lactation.

### 2.3 Interventions and Follow-Up

All eligible patients were divided into high titer group (>150 RU/ml) and non-high titer group (20–150 RU/ml) and subdivided into CTX subgroup and TAC subgroup. Stratified analyses were processed according to different therapeutic models to remove potential confounder in treatment that might cause bias in the association between PLA2Rab levels and outcomes. Therefore, the overall cohort was classified into CTX group and TAC group as well. Patients who received CTX treatment were administered intravenous infusion at 750 mg/m^2^ body surface once every month for 6 months and then once every 2 or 3 months (cumulative dosage, 8–10 g). Subjects treated with TAC were initiated oral TAC on a dose of 0.05–0.1 mg/kg/day (no more than 0.15 mg/kg/day), divided into two equal doses at intervals of 12 h. The dose was adjusted according to the target trough blood concentration of 4–8 ng/ml for the first 6 months and tapered gradually until discontinued at the end of 12 months. Both subgroups were combined with oral glucocorticoids therapy.

The follow-up length was at least 6 months, and the mean follow-up period was 11.70 ± 1.22 months. Clinical parameters levels and remission rates were recorded at the given time (3, 6, and 12 months follow-up). The value of estimated glomerular filtration rate (eGFR) was calculated using the Chronic Kidney Disease Epidemiology Collaboration (CKD-EPI) 2009 creatinine equation (CKD-EPI_2009scr_) (Levey et al., 2009). The detection of serum PLA2Rab was performed using enzyme-linked immunosorbent assay (ELISA) kits (EUROIMMUN, Lübeck, and Germany). According to the manufacturer’s recommendation, a value ≥20 RU/ml was considered positive.

### 2.4 Definitions and Outcomes

Based on the KDIGO 2012 guideline (Beck et al., 2013), (1) complete remission (CR) means that urinary protein is <0.3 g/24 h, accompanied by normal serum albumin and serum creatinine; (2) partial remission (PR), urinary protein <3.5 g/24 h and a 50% or greater reduction from peak values, accompanied by an improvement or normalization of the serum albumin and stable serum creatinine; (3) total remission (TR), a composite remission of CR or PR; (4) relapse, new nephrotic syndrome after an achievement of CR or PR, urinary protein >3.5 g/24 h or >50% of the peak values, and with a reduction in serum albumin; (5) end point of renal survival, compared with baseline, double of serum creatine or a 50% decline in eGFR or progression to end stage renal disease (ESRD) with eGFR <15 ml/min/1.73 m^2^; (6) serious adverse event, any untoward medical incident, including reaching clinical death, significant or permanent disability or incapacity, and life-threatening illness.

Primary outcomes were CR and TR rates at 3, 6, and 12 months follow-up, time to remission. Secondary outcomes were the evolution of urinary protein, serum albumin, serum creatinine and eGFR over time, end point of renal survival, clinical relapse of nephrotic syndrome, and serious adverse event.

### 2.5 Statistical Methods

Data were analyzed with Statistical Product and Service Solutions (SPSS) statistical software for Windows, version 24.0 (SPSS Inc., Chicago, IL, United States) and GraphPad Prism (version 7.0; GraphPad Software, La Jolla, CA). One-sample Kolmogorov–Smirnov testing was used to detect whether variables were normally distributed. Continuous variables with skewed distribution were presented as median (25%–75% interquartile range); categorical variables were presented as frequencies or percentages. Categorical variables were compared with Pearson’s chi-squared (χ^2^) test or Fisher’s exact test. Continuous variables were compared with Mann–Whitney U-test. The correlation between two parameters was analyzed by Spearman’s rank coefficient of correlation. Cumulative probabilities of remission were assessed according to the Kaplan–Meier survival analysis method and the log-rank (Mantel–Cox) test. Univariable and multivariable Cox regression analyses were used to screen for risk factors affecting prognosis. Based on univariate Cox regression analysis and clinical judgements, variables at baseline that might influence the remission with *p* < 0.05 in the univariable analyses were selected into the multivariable Cox regression analysis. Statistical significance was defined as a two-sided *p* < 0.05.

## 3 Results

### 3.1 PLA2Rab Titers With Clinical Baseline Characteristics at Baseline

**FIGURE 1:**
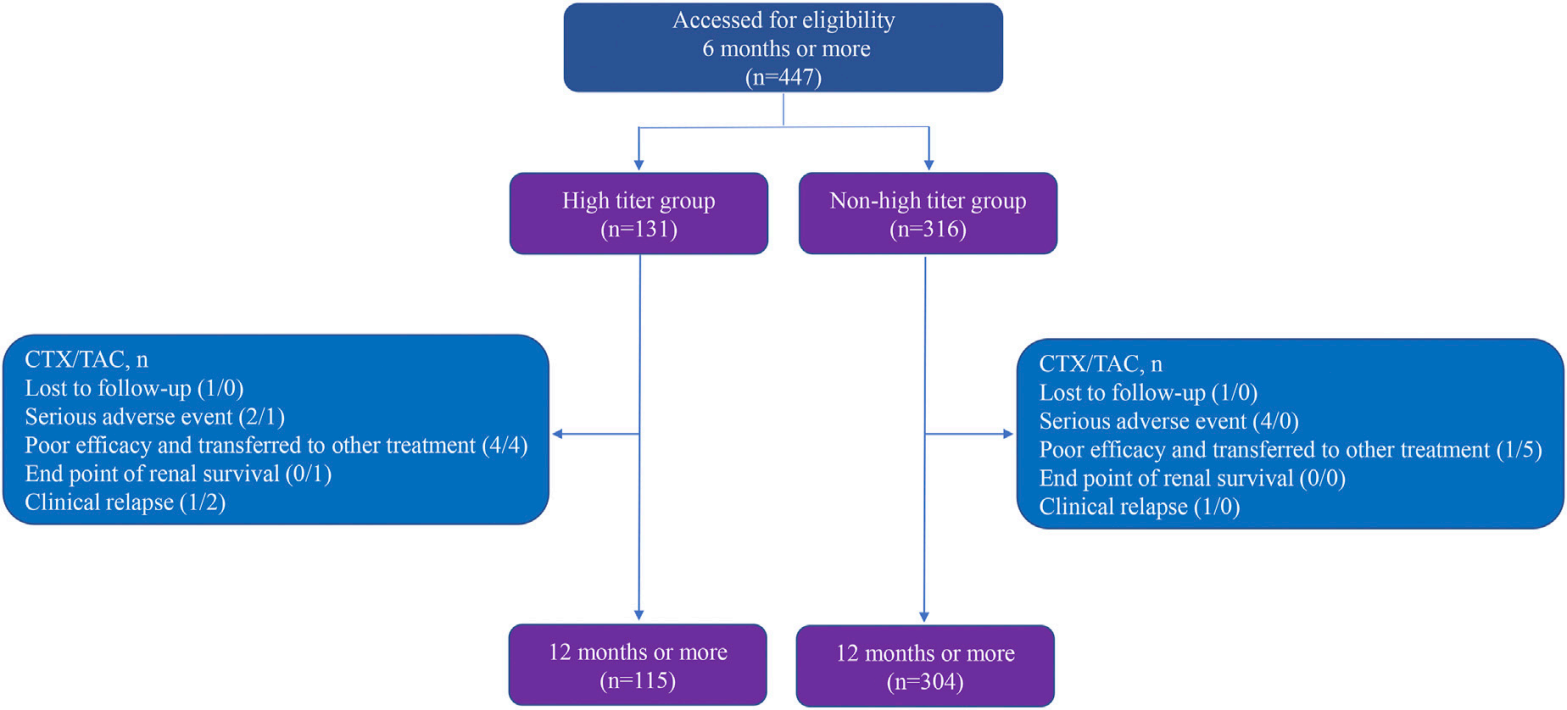
Flow diagram of follow-up for enrollment of participants.

Among 447 patients enrolled, 131 (29.31%) patients were in the high titer group and 316 (70.69%) in the non-high titer group, which include 71 (54.20%) CTX subgroup and 60 (45.80%) TAC subgroup in the high titer group, and 204 (64.56%) CTX subgroup and 112 (35.44%) TAC subgroup in the non-high titer group. A total of 275 (61.52%) patients were in the CTX group and 172 (38.48%) in the TAC group. The overview of follow-up for enrolled patients is described in Figure 1.

We first evaluated the difference in clinical parameters levels at baseline between high titer group and non-high titer group in the overall cohort, CTX group, and TAC group, respectively. Compared to patients with non-high titer antibody, individuals with high titer antibody had significantly higher urinary protein levels and lower serum albumin levels (*p* < 0.001), while the differences in other clinical parameters levels were not statistically significant (Table 1).

**TABLE 1:** Comparison of baseline clinical characteristics between the two titer groups in overall cohort, CTX group, and TAC group.

Parameters	Overall cohort					CTX group				TAC group		
	Total	High titer	Non-high titer	*p*-value	Total	High-titer	Non-high titer	*p*-value	Total	High titer	Non-high titer	*p*-value
	(*n* = 447)	(n = 131)	(*n* = 316)		(*n* = 275)	(*n* = 71)	(*n* = 204)		(*n* = 172)	(*n* = 60)	(*n* = 112)	
Age (years)	54 (44, 64)	55 (47, 63)	53 (42, 64)	0.124	53 (44, 63)	54 (47, 61)	53 (43, 64)	0.394	54 (43, 64)	56 (47, 65)	53 (37, 64)	0.187
Male gender (%)	317 (70.92)	95 (72.52)	222 (70.25)	0.631	198 (72.00)	51 (71.83)	147 (72.06)	0.971	119 (69.19)	44 (73.33)	75 (66.96)	0.389
SBP (mmHg)	129 (120, 139)	130 (122, 139)	129 (120, 140)	0.191	128 (120, 138)	129 (121, 137)	128 (118, 139)	0.379	130 (120, 140)	132 (122, 140)	130 (120, 141)	0.432
DBP (mmHg)	80 (74, 89)	83 (75, 88)	80 (73, 89)	0.079	80 (73, 88)	83 (75, 87)	80 (72, 88)	0.102	82 (74, 90)	83 (72, 92)	81 (74, 90)	0.428
Serum creatinine (µmol/L)	77.89 (63.49, 96.49)	82.20 (64.50, 99.13)	77.44 (63.33, 93.54)	0.186	78.00 (64.10, 97.00)	79.00 (62.63, 98.17)	77.95 (64.34, 94.85)	0.872	77.47 (62.34, 94.95)	85.08 (65.42, 101.63)	75.41 (61.49, 90.95)	0.057
Serum albumin (g/L)	24.87 (20.67, 28.18)	20.62 (18.20, 22.38)	27.10 (23.10, 28.59)	<0.001	25.61 (20.70, 28.30)	20.57 (18.14, 22.80)	26.87 (23.10, 28.51)	<0.001	23.37 (20.42, 28.13)	20.74 (18.43, 22.17)	27.25 (22.99, 28.84)	<0.001
eGFR (ml/min/1.73 m^2^)	93.02 (74.83, 106.83)<	88.93 (75.22, 103.46)	95.41 (73.72, 107.86)	0.067	92.18 (73.26, 106.92)	89.65 (75.90, 105.05)	94.64 (72.87, 107.18)	0.404	94.27 (75.45, 106.64)	88.90 (69.42, 102.44)	96.32 (76.52, 109.91)	0.070
Urinary protein (g/24 h)	7.02 (4.60, 10.37)	11.18 (9.51, 13.66)	5.62 (4.33, 7.78)	<0.001	6.80 (4.55, 10.07)	10.53 (9.12, 14.38)	5.78 (4.30, 7.59)	<0.001	7.90 (4.78, 11.44)	11.69 (10.10, 13.32)	5.51 (4.37, 7.92)	<0.001
TG (mmol/L)	2.37 (1.67, 3.38)	2.48 (1.75, 3.65)	2.33 (1.64, 3.36)	0.271	2.38 (1.64, 3.39)	2.17 (1.59, 3.58)	2.39 (1.64, 3.38)	0.902	2.36 (1.72, 3.37)	2.53 (1.89, 3.86)	2.23 (1.65, 3.25)	0.064
TCHO (mmol/L)	8.20 (6.81, 9.82)	8.51 (7.22, 10.11)	8.03 (6.65, 9.67)	0.081	8.14 (6.83, 9.82)	8.42 (6.91, 10.11)	8.03 (6.80, 9.67)	0.502	8.21 (6.67, 9.81)	8.78 (7.56, 10.15)	7.97 (6.43, 9.64)	0.065
HDL-chol (mmol/L)	1.50 (1.13, 2.01)	1.45 (1.05, 1.98)	1.51 (1.16, 2.05)	0.161	1.59 (1.12, 2.13)	1.55 (1.02, 2.05)	1.60 (1.14, 2.16)	0.323	1.40 (1.14, 1.88)	1.34 (1.07, 1.87)	1.48 (1.16, 1.88)	0.384
LDL-chol (mmol/L)	4.73 (3.58, 5.95)	4.99 (3.91, 6.14)	4.61 (3.50, 5.90)	0.063	4.71 (3.58, 6.12)	4.84 (3.73, 6.35)	4.61 (3.58, 5.90)	0.378	4.77 (3.58, 5.91)	5.03 (4.25, 6.06)	4.61 (3.14, 5.91)	0.092
PLA2R titer (RU/ml)	87.59 (39.48, 200.00)	280.61 (226.79, 360.18)	59.02 (31.83, 95.10)	<0.001	80.70 (37.33, 160.50)	280.61 (215.70, 356.63)	61.35 (32.00, 99.92)	<0.001	94.60 (42.67, 240.58)	281.44 (231.87, 370.74)	52.1 (30.15, 92.18)	<0.001

Data were shown as median (25–75% interquartile range). *p*-value: high titer vs. non-high titer. *p*-value <0.05 was considered statistically significant. CTX, cyclophosphamide; TAC, tacrolimus; SBP, systolic blood pressure; DBP, diastolic blood pressure; eGFR, estimated glomerular filtration rate; TG, triglyceride; TCHO, total cholesterol; HDL-chol, high-density lipoprotein cholesterol; LDL-chol, low-density lipoprotein cholesterol; PLA2Rab, antibodies against the M-type phospholipase-A2-receptor

**FIGURE 2:**
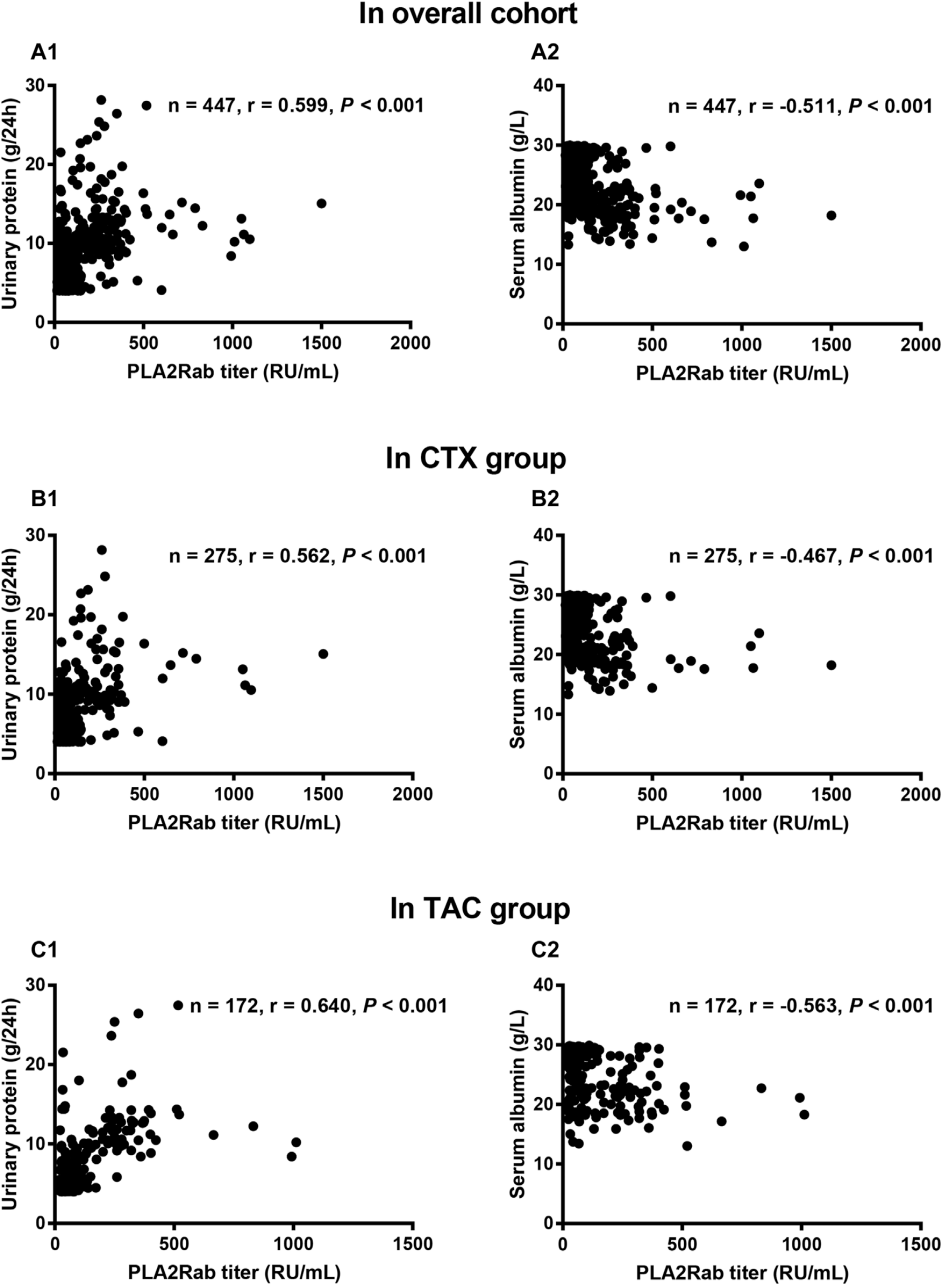
Correlation analyses between serum PLA2Rab titer and urinary protein and serum albumin. The serum PLA2Rab titer positively associated with urinary protein in overall cohort **(A1)**, CTX group **(B1)**, and TAC group **(C1)**; but negatively associated with serum albumin in overall cohort **(A2)**, CTX group **(B2)**, and TAC group **(C2)**. CTX: cyclophosphamide; TAC: tacrolimus; PLA2Rab: antibody against the M-type phospholipase-A2-receptor.

We next assessed the correlations between PLA2Rab and clinical parameters at baseline. In the overall cohort, CTX group, and TAC group, correlation analysis indicated that antibody titer positively correlated with urinary protein level (*r* = 0.599, 0.562, and 0.640, respectively, all *p* < 0.001) and negatively correlated with serum albumin level (*r* = −0.511, −0.467, and −0.563, respectively, all *p* < 0.001) (Figure 2), while the correlations with other clinical parameters were either non-existent or very weak (Table 2).

**TABLE 2:** Correlation analysis between PLA2Rab and clinical parameters in overall cohort, CTX group, and TAC group.

Parameters	Overall cohort		CTX group		TAC group	
	CO	*p* value	CO	*p*-value	CO	*p*-value
Age (years)	0.096	0.043	0.034	0.578	0.177	0.020
Male gender	0.039	0.413	0.074	0.223	0.005	0.943
SBP (mmHg)	0.077	0.104	0.039	0.518	0.116	0.129
DBP (mmHg)	0.105	0.027	0.112	0.063	0.090	0.242
Serum creatinine (µmol/L)	0.092	0.052	0.053	0.380	0.150	0.049
eGFR (mL/min/1.73 m^2^)	−0.110	0.020	−0.047	0.439	−0.194	0.011
TG (mmol/L)	0.122	0.010	0.106	0.080	0.147	0.054
TCHO (mmol/L)	0.110	0.020	0.089	0.142	0.144	0.059
HDL-chol (mmol/L)	−0.051	0.281	−0.041	0.496	−0.078	0.307
LDL-chol (mmol/L)	0.075	0.111	0.106	0.080	0.035	0.653

PLA2Ra, antibodies against the M-type phospholipase-A2-receptor; CTX, cyclophosphamide; TAC, tacrolimus; CO, correlation coefficient; SBP, systolic blood pressure; DBP: diastolic blood pressure; eGFR, estimated glomerular filtration rate; TG, triglyceride; TCHO, total cholesterol; HDL-chol, high-density lipoprotein cholesterol; LDL-chol, low-density lipoprotein cholesterol. *p* < 0.05 was considered significant difference.

### 3.2 Primary Outcome

**FIGURE 3:**
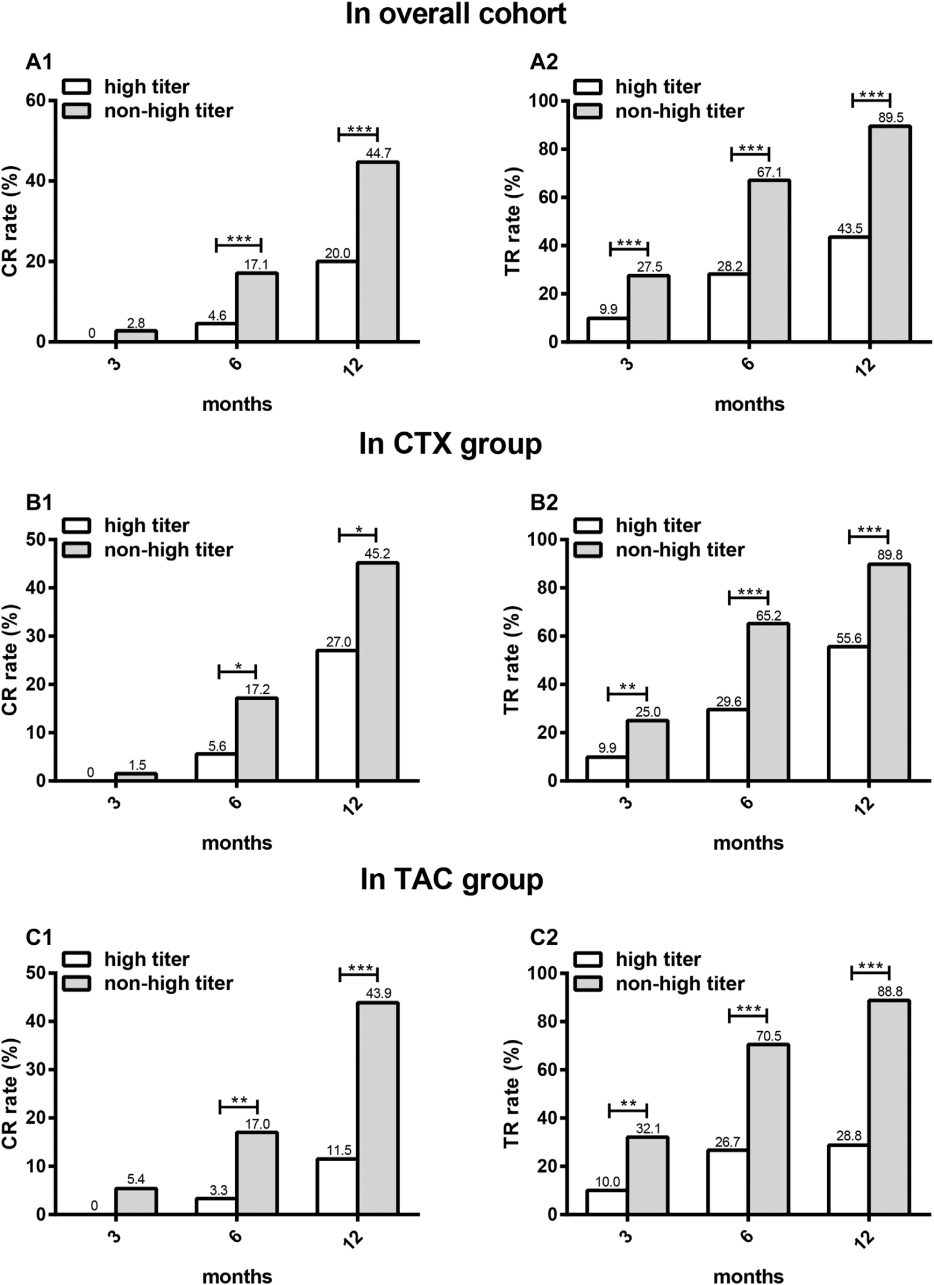
The comparison of CR and TR rates between high titer group and non-high titer group at different time points in different patients. CR **(A1)** and TR **(A2)** in overall cohort, CR **(B1)** and TR **(B2)** in CTX group, CR **(C1)** and TR **(C2)** in TAC group. CR: complete remission; TR: total remission; CTX: cyclophosphamide; TAC: tacrolimus. *p < 0.05, **p < 0.01, ***p < 0.001.

In overall cohort, CTX group, and TAC group, CR occurred in the high titer group significantly less frequently compared with the non-high titer group at 6 and 12 months (*p* < 0.001). There was a nonsignificant trend for lower CR in the high titer group than in the non-high titer group at 3 months (*p* = 0.064). Then, the same analyses were performed on TR. TR occurred in the high titer group significantly less frequently compared to the non-high titer group at 3, 6, and 12 months (*p* < 0.001) (Figure 3).

**FIGURE 4:**
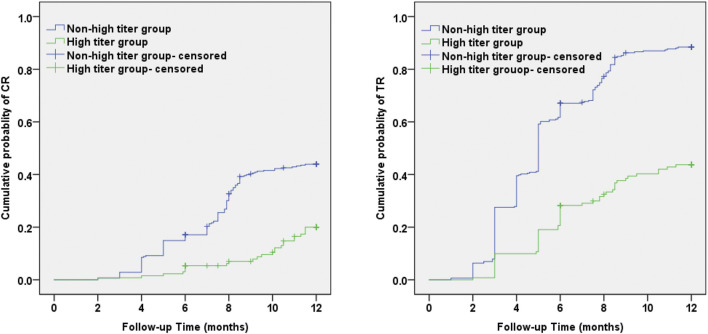
Kaplan–Meier survival analyses of antibody against M-type phospholipase-A2-receptor level and cumulative probability of CR and TR during the 12 months observation period. Incidence of CR and TR over time was significantly higher in the non-high titer group than in the high titer group (Log-Rank, *p* < 0.001). CR: complete remission; TR: total remission.

The cumulative incidences of CR and TR using the Kaplan–Meier method and log-rank test were significantly lower in the high titer group compared with the non-high titer group (χ^2^ = 24.02, *p* < 0.001 and 91.97, *p* < 0.001, respectively) (Figure 4). The average time to CR and TR in the high titer group was 11.36 ± 0.15 months and 9.42 ± 0.29 months, significantly longer than CR (9.67 ± 0.17 months) and TR (5.82 ± 0.17 months) in the non-high titer group.

Univariate Cox regression analysis of CR showed that baseline levels of PLA2Rab, urinary protein, and serum albumin, and diastolic blood pressure were predictive factors for achieving CR. Then, we performed the same analyses on TR. Univariate Cox regression analysis of TR showed that baseline levels of PLA2Rab titer, serum creatinine, eGFR, serum albumin, urinary protein, and age were predictive factors for achieving TR. Multivariable analysis indicated that PLA2Rab level at baseline—besides baseline level of serum albumin—was an independent predictive factor of CR and TR (Table 3).

**TABLE 3:** Factors predicting CR and TR for patients with seropositive PLA2R-associated MN.

Variables	CR						TR					
	Univariate analysis			Multivariate analysis			Univariate analysis			Multivariate analysis		
	HR	95%CI	*p*-value	HR	95%CI	*p*-value	HR	95%CI	*p*-value	HR	95%CI	*p*-value
Treatment	0.799	0.576, 1.109	0.180	—	—	—	0.822	0.654, 1.032	0.091	—	—	—
PLA2Rab titer (RU/ml)	0.996	0.994, 0.997	**<0.001**	0.997	0.995, 0.999	**0.001**	0.996	0.995, 0.997	**<0.001**	0.997	0.996, 0.998	**<0.001**
Age (years)	0.989	0.978, 1.001	0.066	—	—	—	0.989	0.981, 0.997	**0.009**	0.990	0.976, 1.003	0.142
Gender	0.868	0.621, 1.213	0.407	—	—	—	0.834	0.660, 1.054	0.129	—	—	—
SBP (mmHg)	0.993	0.983, 1.003	0.157	—	—	—	0.995	0.988, 1.001	0.126	—	—	—
DBP (mmHg)	0.984	0.970, 1.000	**0.044**	0.990	0.974, 1.005	0.181	0.991	0.981, 1.001	0.079	—	—	—
Serum creatinine (µmol/L)	0.995	0.989, 1.002	0.166	—	—	—	0.994	0.990, 0.999	**0.020**	0.993	0.981, 1.006	0.291
Serum albumin (g/L)	1.109	1.065, 1.155	**<0.001**	1.083	1.012, 1.159	**0.021**	1.110	1.080, 1.141	**<0.001**	1.094	1.045, 1.146	**<0.001**
eGFR (mL/min/1.73 m^2^)	1.006	0.999, 1.014	0.790	—	—	—	1.007	1.002, 1.011	**0.009**	0.994	0.978, 1.009	0.421
Urinary protein (g/24 h)	0.908	0.866, 0.951	**<0.001**	1.033	0.962, 1.109	0.371	0.913	0.886, 0.942	**<0.001**	1.042	0.995, 1.091	0.078
TG (mmol/L)	1.007	0.935, 1.085	0.853	—	—	—	0.987	0.934, 1.044	0.654	—	—	—
TCHO (mmol/L)	1.002	0.938, 1.070	0.959	—	—	—	0.985	0.938, 1.035	0.550	—	—	—
HDL-chol (mmol/L)	0.937	0.720, 1.219	0.629	—	—	—	1.008	0.839, 1.212	0.929	—	—	—
LDL-chol (mmol/L)	0.986	0.913, 1.064	0.719	—	—	—	0.962	0.910, 1.018	0.180	—	—	—

Variables with *p* < 0.20 in the univariable analyses were included in the multivariable analysis. CR, complete remission; TR, total remission; PLA2R, M type phospholipase A2 receptor; MN, membranous nephropathy; HR: hazard ratio; CI, confidence interval; PLA2Rab: antibodies against the M-type phospholipase-A2-receptor; SBP, systolic blood pressure; DBP, diastolic blood pressure; eGFR, estimated glomerular filtration rate; TG, triglyceride; TCHO, total cholesterol; HDL-chol: high-density lipoprotein cholesterol; LDL-chol, low-density lipoprotein cholesterol. The meaning of the bold values is highlight *p* < 0.05.

**FIGURE 5:**
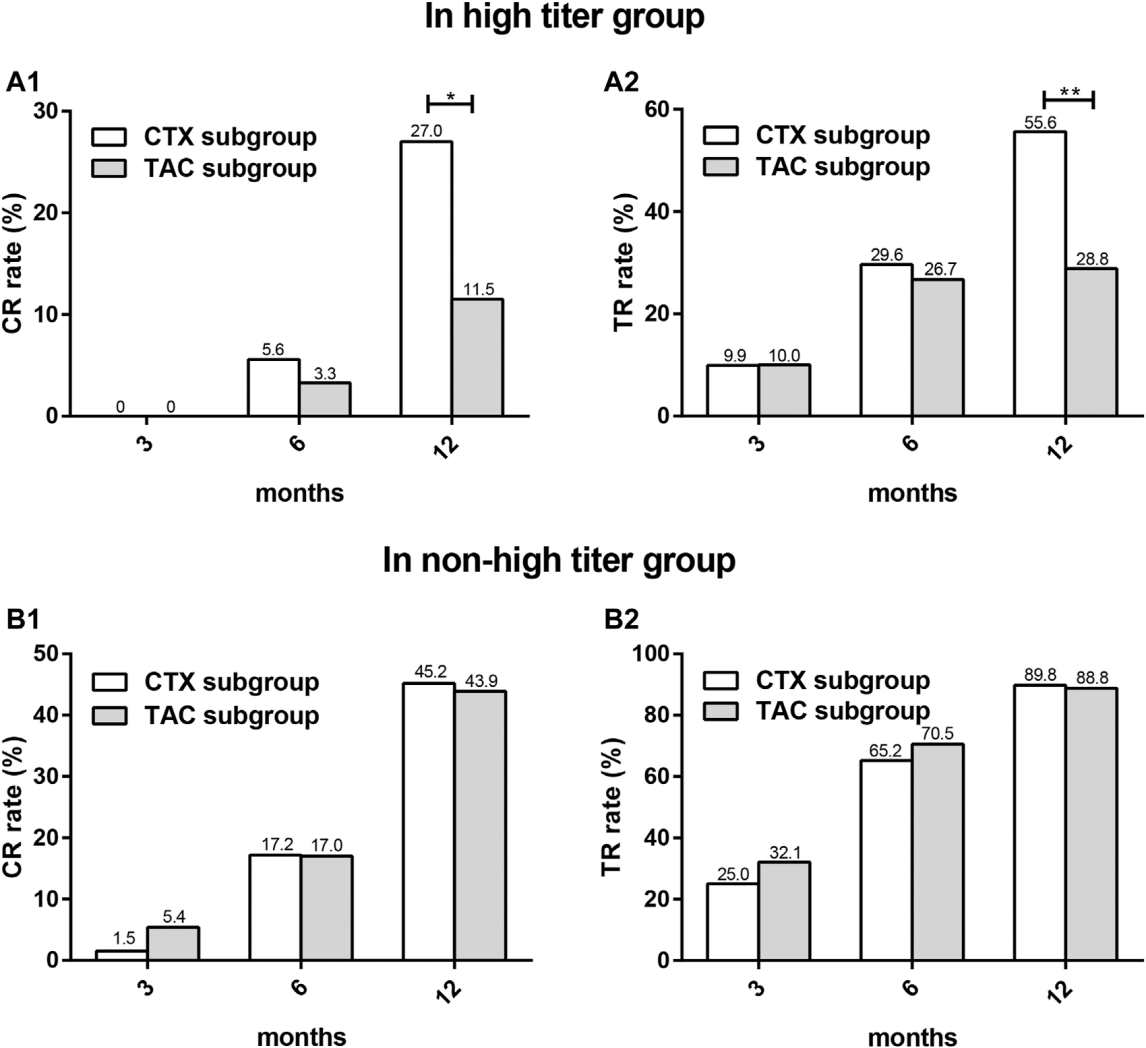
The comparison of CR and TR rates between CTX subgroup and TAC subgroup at different time points in two titer groups. CR **(A1)** and TR **(A2)** in high titer group, CR **(B1)** and TR **(B2)** in non-high titer group.CR: complete remission; TR: total remission; CTX: cyclophosphamide; TAC: tacrolimus. *p < 0.05, **p < 0.01.

We next evaluated the differences in remission rates between the CTX subgroup and the TAC subgroup in the two titer groups, respectively. There were no significant differences in the baseline levels of clinical parameters between the two subgroups (Table 4). In the high titer group, CR and TR occurred in the CTX subgroup significantly more frequently compared with that in the TAC subgroup at 12 months (*p* = 0.039 and 0.004, respectively), while in the non-high titer group, and there was a nonsignificant trend for higher CR and TR in the CTX subgroup than in the TAC subgroup at 12 months (*p* = 0.834 and 0.773, respectively) (Figure 5).

**TABLE 4:** Comparison of baseline clinical characteristics between CTX subgroup and TAC subgroup in the two titer groups.

Parameters	High titer group					Non-high titer group		
	Total	CTX subgroup	TAC subgroup	*p*-value	Total	CTX subgroup	TAC subgroup	*p*-value
	(*n* = 131)	(*n* = 71)	(*n* = 60)		(*n* = 316)	(*n* = 204)	(*n* = 112)	
Age (years)	55 (47, 63)	54 (47, 61)	56 (47, 65)	0.571	42 (53, 64)	53 (43, 64)	53 (37, 64)	0.555
Male gender (%)	95 (72.52)	51 (71.83)	44 (73.33)	0.848	222 (70.25)	147 (72.06)	75 (66.96)	0.344
SBP (mmHg)	130 (122, 139)	129 (121, 137)	132 (122, 140)	0.287	129 (120, 140)	128 (118, 139)	130 (120, 141)	0.117
DBP (mmHg)	83 (75, 88)	83 (75, 87)	83 (72, 92)	0.897	80 (73, 89)	80 (72, 88)	81 (74, 90)	0.244
Serum creatinine (µmol/L)	82.20 (64.50, 99.13)	79.00 (62.63, 98.17)	85.08 (65.42, 101.63)	0.499	77.44 (63.33, 93.54)	77.95 (64.34, 94.85)	75.41 (61.49, 90.95)	0.159
Serum albumin (g/L)	20.62 (18.20, 22.38)	20.57 (18.14, 22.80)	20.74 (18.43, 22.17)	0.895	27.10 (23.10, 28.59)	26.87 (23.10, 28.51)	27.25 (22.99, 28.84)	0.799
eGFR (ml/min/1.73 m^2^)	88.93 (75.22, 103.46)	89.65 (75.90, 105.05)	89.18 (72.32, 101.82)	0.695	95.41 (73.72, 107.86)	94.64 (72.87, 107.18)	96.32 (76.52, 109.91)	0.289
Urinary protein (g/24 h)	11.18 (9.51, 13.66)	10.53 (9.12, 14.38)	11.70 (10.10, 13.32)	0.149	5.62 (4.33, 7.78)	4.30 (5.78, 7.59)	5.51 (4.37, 7.92)	0.926
TG (mmol/L)	2.48 (1.75, 3.65)	2.17 (1.59, 3.58)	2.53 (1.89, 3.86)	0.231	2.33 (1.64, 3.36)	2.39 (1.64, 3.38)	2.23 (1.65, 3.25)	0.506
TCHO (mmol/L)	8.51 (7.22, 10.11)	8.42 (6.91, 10.11)	8.78 (7.56, 10.15)	0.451	8.03 (6.65, 9.67)	8.03 (6.80, 9.67)	7.97 (6.43, 9.64)	0.455
HDL-chol (mmol/L)	1.45 (1.05, 1.98)	1.55 (1.02, 2.05)	1.34 (1.07, 1.87)	0.371	1.51 (1.16, 2.05)	1.60 (1.14, 2.16)	1.48 (1.16, 1.88)	0.090
LDL-chol (mmol/L)	4.99 (3.91, 6.14)	4.84 (3.73, 6.35)	5.03 (4.25, 6.06)	0.666	4.61 (3.50, 5.90)	4.61 (3.58, 5.90)	4.61 (3.14, 5.91)	0.509
PLA2R titer (RU/ml)	280.61 (226.79, 360.18)	280.61 (215.70, 356.63)	281.44 (231.87, 370.74)	0.705	59.02 (31.83, 95.10)	61.35 (32.00, 99.92)	52.10 (30.15, 92.18)	0.439

Data were showed as median (25%–75% interquartile range). *p* value: CTX, subgroup vs. TAC subgroup. *p*-value <0.05 was considered statistically significant. CTX: cyclophosphamide; TAC: tacrolimus; SBP: systolic blood pressure; DBP: diastolic blood pressure; eGFR: estimated glomerular filtration rate; TG: triglyceride; TCHO: total cholesterol; HDL-chol: high-density lipoprotein cholesterol; LDL-chol: low-density lipoprotein cholesterol; PLA2Rab: antibodies against the M-type phospholipase-A2-receptor.

### 3.3 Secondary Outcomes

#### 3.3.1 Evolution of Clinical Parameters

**FIGURE 6:**
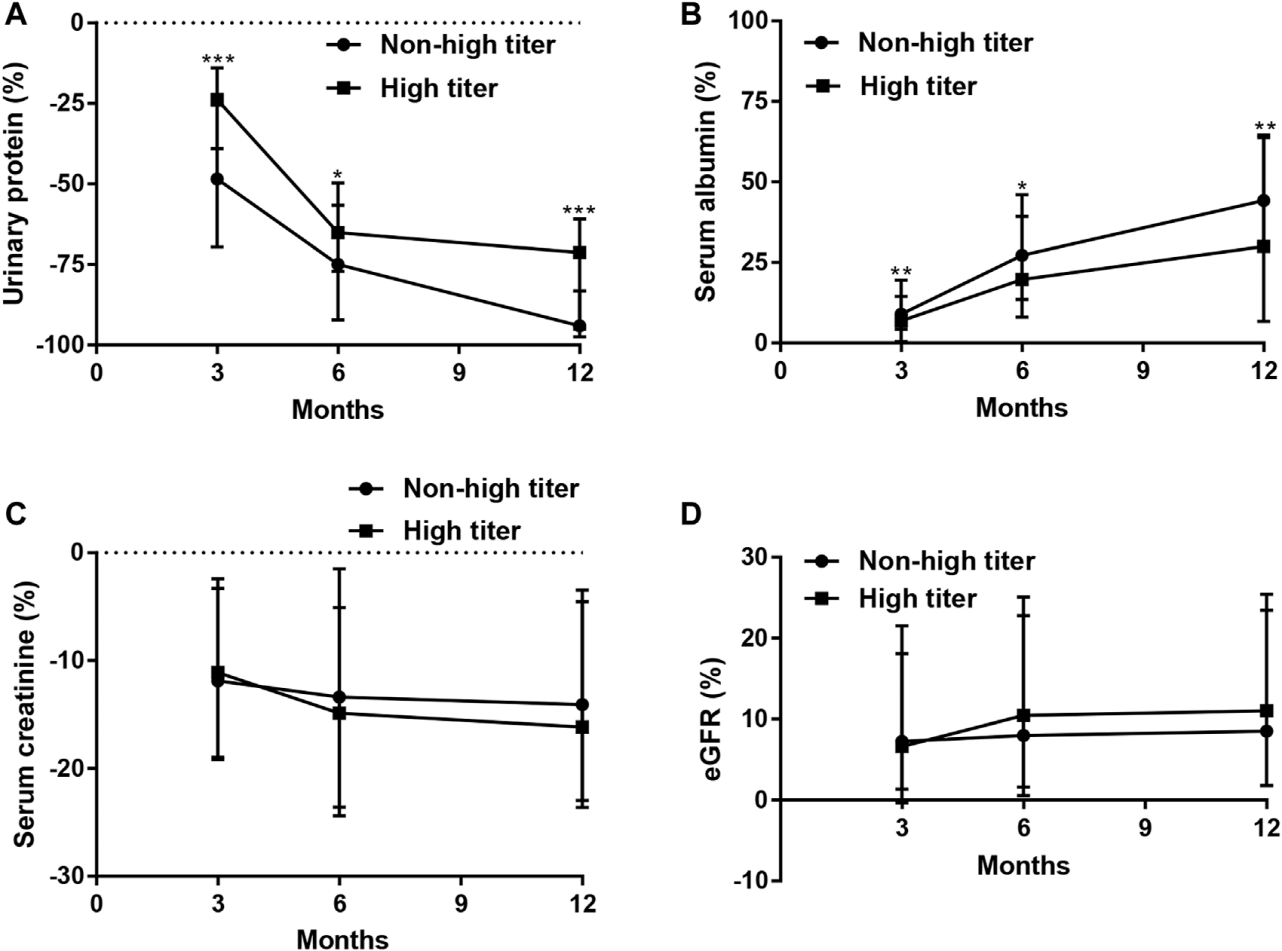
Percentage changes in urinary protein, serum albumin, serum creatinine, and eGFR over time. Compared to baseline, at 3, 6, and 12 months, the decrease of urinary protein **(A)** (*p* < 0.001, *p* = 0.016, *p* < 0.001, respectively) and the increase of serum albumin **(B)** (*p* = 0.002, 0.016, 0.001, respectively) more rapid in non-high titer group than in high titer group, while there were no significant differences in the decrease in serum creatinine **(C)** (*p* = 0.825, 0.499, 0.440, respectively) and in the increase in eGFR **(D)** (*p* = 0.971, 0.291, 0.460, respectively) at 3, 6, and 12 months between the two titer groups. eGFR: estimated glomerular filtration rate. *p < 0.05, **p < 0.01, ***p < 0.001.

At 3, 6, and 12 months, compared to the non-high titer group, the high titer group had significantly higher urinary protein and lower serum albumin (*p* < 0.001), while it had a nonsignificant trend for higher serum creatinine and lower eGFR (*p* > 0.05). Moreover, at the given time, the percentage improvement in urinary protein and serum albumin was less significant in the high titer group than in the non-high titer group (*p* < 0.05); however, the changes in serum creatinine and eGFR were not significant (*p* > 0.05) (Table 5; Figure 6).

**TABLE 5:** The comparison of clinical parameters over time.

	Clinical parameters values					Percentage changes of clinical parameters values			
Parameter	Months	Overall cohort	High titer	Non-high titer	*p*-value	Overall cohort	High titer	Non-high titer	*p*-value
Up (g/24 h)	Baseline	7.02 (4.60, 10.37)	11.18 (9.51, 13.66)	5.62 (4.33, 7.78)	<0.001	—	—	—	—
	3	3.61 (3.01, 6.25)	8.72 (6.51, 10.57)	3.52 (2.27, 3.69)	<0.001	−40.77 (−62.94, -20.87)	−23.82 (−39.06, −14.00)	−48.44 (−69.58, −25.53)	<0.001
	6	2.22 (0.68, 3.95)	3.84 (2.94, 4.60)	1.41 (0.46, 3.52)	<0.001	−69.65 (−89.54, −53.78)	−65.10 (−77.09, −56.66)	−74.95 (−92.17, −49.73)	0.016
	12	0.51 (0.20, 2.41)	3.54 (0.55, 4.13)	0.34 (0.15, 1.03)	<0.001	−92.13 (−97.19, −71.99)	−71.29 (−95.05, −60.86)	−94.07 (−97.50, −83.20)	<0.001
Salb (g/L)	Baseline	24.87 (20.67, 28.18)	20.62 (18.20, 22.38)	27.10 (23.10, 28.59)	<0.001	—	—	—	—
	3	27.15 (22.56, 31.03)	20.94 (20.01, 24.30)	30.08 (25.61, 31.50)	<0.001	8.54 (3.61, 17.79)	6.88 (0.46, 14.46)	8.96 (4.32, 19.52)	0.002
	6	32.74 (25.15, 36.12)	24.13 (21.30, 30.60)	34.03 (30.32, 37.30)	<0.001	25.88 (11.64, 44.01)	19.71 (8.04, 39.25)	27.21 (13.54, 46.01)	0.016
	12	36.10 (31.75, 40.20)	27.18 (21.04, 34.71)	39.00 (34.81, 41.17)	<0.001	41.76 (23.43, 63.81)	29.94 (6.70, 63.81)	44.22 (30.79, 64.56)	0.001
Scr (µmol/L)	Baseline	77.89 (63.49, 96.49)	82.20 (64.50, 99.13)	77.44 (63.33, 93.54)	0.186	—	—	—	—
	3	67.70 (60.12, 80.57)	69.57 (60.15, 82.50)	66.50 (60.04, 80.45)	0.329	−11.54 (−19.04, −2.81)	−11.11 (−18.97, −2.42)	−11.89 (−19.16, −3.31)	0.825
	6	65.48 (56.90, 75.69)	68.59 (58.19, 78.30)	64.30 (56.58, 75.46)	0.212	−13.95 (−23.84, −3.02)	−14.88 (−23.59, −5.08)	−13.40 (−24.39, −1.48)	0.499
	12	66.13 (58.97, 77.13)	66.32 (60.00, 78.25)	65.97 (58.23, 76.22)	0.351	−14.52 (−23.49, −3.95)	−16.16 (−22.96, −4.54)	−14.09 (−23.62, −3.47)	0.440
eGFR (ml/min/1.73 m^2^)	Baseline	93.02 (74.83, 106.83)	88.93 (75.22, 103.46)	95.41 (73.72, 107.86)	0.067	—	—	—	—
	3	100.40 (87.40, 111.21)	97.74 (84.73, 109.03)	102.36 (87.69, 111.66)	0.114	7.19 (1.14, 19.25)	6.61 (−0.34, 21.52)	7.25 (1.35, 18.08)	0.971
	6	101.87 (91.10, 111.56)	101.23 (88.26, 108.87)	102.11 (92.08, 113.00)	0.125	9.14 (0.78, 23.54)	10.44 (1.60, 25.09)	7.99 (0.54, 22.79)	0.291
	12	102.36 (91.72, 111.09)	101.03 (88.83, 109.68)	102.65 (92.08, 111.59)	0.153	9.39 (1.86, 23.91)	11.00 (1.78, 25.40)	8.53 (1.81, 23.44)	0.460

Data were showed as median (25%–75% interquartile range). *p*-value: high titer vs. non-high titer. *p*-value <0.05 was considered statistically significant. Up, urinary protein; Salb, serum albumin; Scr, serum creatinine; eGFR, estimated glomerular filtration rate.

#### 3.3.2 End Point of Renal Survival and Clinical Relapse

By the end of the follow-up period, renal function remained stable in the majority of the subjects. Only one patient who received TAC treatment reached the end point of renal survival in the high titer group, who had no remission of proteinuria. Compared to non-high titer group, a nonsignificant trend for higher incidence of the end point of renal survival in the high titer group was observed during the observation period (*p* = 0.293).

Clinical relapses were recorded 3 (CTX/TAC subgroup: 1/2) out of 131 patients (2.29%) in the high titer group and 1 (CTX/TAC subgroup: 1/0) out of 316 subjects (0.32%) in the non-high titer group, who had achieved PR prior to clinical relapse during the 12 months follow-up period (*p* = 0.078).

#### 3.3.3 Serious Adverse Event

Seven severe infections were observed in 3 (CTX/TAC subgroup: 2/1) out of 131 patients (2.29%) in the high titer group and 4 (CTX/TAC subgroup: 4/0) out of 316 subjects (1.27%) in the non-high titer group (*p* = 0.423). In the high titer group, of the two patients treated with CTX, one developed respiratory failure caused by severe pneumocystis pneumonia, and the other suffered from fatal *Staphylococcus aureus* sepsis, and one patient who received TAC treatment had necrosis of the femoral head. Similarly, in the non-high titer group, four individuals treated with CTX also developed respiratory failure caused by severe pneumonia, including three pneumocystis pneumonia and one pneumocystis combined with bacterial pneumonia. In addition, one patient who received TAC treatment experienced severe cardiovascular event in the high titer group, which was perhaps unrelated to the treatment, as he had a previous history of cardiovascular disease. None of the patients developed thrombosis or thromboembolic events, new tumors, and clinical deaths.

## 4 Discussion

In this cohort, we first investigated the clinical relevance of PLA2Rab by assessing the association of PLA2Rab with clinical parameters at baseline. Serum albumin and urinary protein are generally used to quantitatively evaluate the severity of nephrotic syndrome in MN, which are important indicators for assessing disease activity (Kanigicherla et al., 2013; Lin et al., 2015; Gopalakrishnan et al., 2016; Li et al., 2016). Consistent with data from aforementioned studies (Li et al., 2016; Song et al., 2018; Provatopoulou et al., 2019; Katsumata et al., 2020; Li et al., 2021), we found a significant association of PLA2Rab with urinary protein and serum albumin indicating the correlation between antibody levels and disease activity (Mahmud et al., 2019; Gong et al., 2020; Guo et al., 2020). However, Jullien et al. (2017) claimed that the initial PLA2Rab level did not relate to the degree of proteinuria. It is likely that the distinction may be due, in part, to genetic or racial or environmental background difference.

Additionally, previous studies showed that higher PLA2Rab levels were related to higher levels of serum creatinine and lower eGFR (Hofstra et al., 2011; Provatopoulou et al., 2019), which was inconsistent with the findings of our study. The reason for this discrepancy may be associated with the facts that the kidney function of the subjects in our study was within the normal range or mildly abnormal.

It is known that binding of PLA2Rab in circulation and PLA2R antigen on glomerular podocyte to form an *in situ* immune complex activates a complement to result in podocyte and immune-mediated injury, ultimately causing urinary protein production (Beck et al., 2009; Glassock, 2012; Mcquarrie, 2018). It appears reasonable to speculate that a higher level of PLA2Rab in circulation might result in increased binding to PLA2R on podocyte, more subepithelial deposition of antibody–antigen immune complexes, which lead to more serious damage of podocyte and the destruction of the filtration barrier, and eventually increased levels of proteinuria (Kerjaschki, 2004; Hofstra et al., 2011; Song et al., 2018).

Next, we assessed the differences in remission rates between the two titer groups. CR and TR rates in overall cohort, CTX group, and TAC group were significantly lower in the high titer group than in the non-high titer group, which suggests that the PLA2Rab levels correlate with the rate of clinical remission (Pourcine et al., 2017; Zhang et al., 2017; Han et al., 2019). In the GEMRITUX Study, PLA2Rab titer of <275 RU/ml at baseline was significantly associated with a composite end point of CR or PR (Dahan et al., 2017). Ruggenenti et al. (2015) reported that, compared to those in the highest titer (>204 RU/ml), the probability of achieving the composite remission of CR or PR was approximately four- and twofold higher in the lowest (14–86 RU/ml) and middle (87–204 RU/ml) titer, respectively; compared to the highest one, the probability of achieving CR was approximately threefold higher in the lowest and middle titer. The study of Ramachandran et al. (2016) also showed that a greater proportion of patients in the first two tertiles [(20.54–135.90) RU/ml, (149.23–335.18) RU/ml, respectively] had CR compared to individuals in the third tertile (337.16–6,528) RU/ml. Moreover, updated meta-analyses revealed that serum PLA2Rab level at baseline was closely associated with the clinical remission (Rao et al., 2020). Of interest, these conclusion held as well when limiting the analysis to the CTX group and TAC group, which have not been reported so far.

Kaplan–Meier survival analysis and log-rank test also showed that the cumulative probability of CR and TR were significantly lower and remarkably slower in the high titer group than in the non-high titer group at 12 months follow-up, which were basically in agreement with the findings of initial studies (Qin et al., 2011; Hofstra et al., 2012; Hoxha et al., 2014a). In the study by Ruggenenti et al. (2015), over a median follow-up of 30.8 months, time to the composite remission of CR or PR progressively increased from the lowest (14–86 RU/ml) to the middle (87–204 RU/ml) and the highest titer (>204 RU/ml) [5.4 (4.1–8.8), 9.1 (3.5–16.4), and 11.6 (5.3–24.8)]. To exclude influence of other baseline parameters, we performed multivariate Cox regression analysis. Adjusted for age, gender, blood pressure, and other clinical parameters, multivariate Cox regression analysis demonstrated that PLA2Rab level at baseline was an independent predictive factor for achieving CR and TR. Baseline PLA2Rab level can be regarded as an important prognostic factor in primary MN (Kim et al., 2015; Fiorentino et al., 2016; Song et al., 2018).

Unfortunately, in our study, the median time to CR and TR was not obtained due to more than half of patients did not achieve CR and TR in the high titer group, and mean time was described instead of median time. It is possible that “lack of remission” was, in part, because of the nature of chronic course of MN and inadequate follow-up time in 12 months follow-up period. Patients may continue to achieve remission within 12–18 months after completion of the initial treatment regimen (Beck et al., 2013). If the follow-up period is extended, more differences between the two titer groups might be found. Of course, on the other hand, this indicated that the prognosis of patients with high PLA2Rab titer was worse, which was also supported by another result in our study that compared with the non-high titer group, and percentage increase in serum albumin and decrease in urinary protein were significantly lower in the high titer group. It is likely that due the fact that serum PLA2Rab level was associated with disease activity, the lower remission rates and slower response in the high titer group might be due to the relatively high activity of the disease (Ruggenenti et al., 2015).

Of note, in the high titer group, the proportions of CR and TR at 12 months in the CTX subgroup were significantly higher than those in the TAC subgroup (CR, 27.0 and 11.5%, *p* = 0.039, TR, 55.6 and 28.8%, and *p* = 0.004, respectively), while in the non-high titer group, there was a nonsignificant trend for higher CR and TR in the CTX subgroup (CTX/TAC, CR, 45.2 and 43.9%, *p* = 0.834, TR, 89.8 and 88.8%, and *p* = 0.773, respectively), which appeared to suggest that CTX is preferred for patients with high PLA2Rab levels. The 2020 KDIGO draft guidelines recommend PLA2Rab level above 150 RU/ml as one of the clinical criteria for high risk of progressive kidney function loss and CTX combined with corticosteroids for patients at high risk of progression. A study by Van de Logt et al. showed that no difference was observed in immunological remission rate between rituximab or cyclophosphamide treatments in patients with low (15–84 RU/ml) or moderate (85–151 RU/ml) antibody levels. In contrast, in patients with high (152–776 RU/ml) antibody levels, cyclophosphamide was more effective than rituximab in inducing an immunological remission, and both baseline antibody level and type of therapy were significantly independent predictors, which suggests that the detection of PLA2Rab might be of great value, particularly for the guidance of initial immunosuppressive therapy (van de Logt et al., 2018).

There was a nonsignificant trend for higher incidences of clinical relapse in the high titer group than in the non-high titer group (2.29% and 1.27%, respectively), which was significantly lower than that reported in initial studies. This result might be limited by a relatively short follow-up time. A previous study reported that baseline anti-PL2R levels were not associated with the appearance of clinical relapses (Provatopoulou et al., 2019). However, Mahmud et al. (2019) found that high PLA2Rab level at baseline was a risk factor for clinical relapse. In addition, in our study, there was a nonsignificant trend for higher incidence of the end point of renal survival in the high titer group than in the non-high titer group, whereas Kanigicherla et al. (2013) reported that the risk of doubling of serum creatinine increased remarkably in patients with high PLA2Rab level. These differences may be explained by discrepancies in genetic, environmental condition, ethnic background, study design, state and stage of clinical disease when patient was enrolled, and clinical treatment method.

In the end, we analyzed serious adverse event. There was a nonsignificant trend more likely to occur in the high titer group than in the non-high titer group. It is worth noting that in six patients who received CTX plus glucocorticoids, serious infections occurred. A remarkable and rather unexpected finding of this study was that only one serious adverse event was noted in TAC group. In addition, it is hard to draw definitive conclusions on the risk of cancer within the limited follow-up time.

Even though validation at five different independent institutions without much discrepancy would increase the strength of this study, the foremost limitation of the present study is the retrospective character, which cannot control all potential confounders that might cause bias. Second, a 12-month follow-up time was relatively short. Admittedly, in a relatively short-term follow-up period, the analyses on clinical relapse and the end point of renal survival were limited. Third, all patients were Chinese, which might restrict the applicability to other ethnic groups. Therefore, in future research with a larger dataset and different subset of patients, prospective randomized design with an extended follow-up period should be performed to validate the established relationships and further study the outcomes after 12 months of immunosuppressive treatments.

In conclusion, high-risk rank patients with PLA2Rab level above 150 RU/ml have higher disease activity and worse prognosis among patients with seropositive PLA2R-associated MN, even under different immunosuppressive therapeutic models; moreover, CTX combined with corticosteroids was preferred compared to TAC plus corticosteroids, although serious adverse events were more frequent in the former. In addition, baseline PLA2Rab level was an independent predictive factor for clinical remission.

## Data Availability

The raw data supporting the conclusion of this article will be made available by the authors, without undue reservation.
